# Optimizing Paediatric Hypospadias Surgical Repair: Pudendal Nerve Block Versus Caudal Block for Superior Analgesia

**DOI:** 10.4274/TJAR.2025.241773

**Published:** 2025-05-30

**Authors:** Hemachander Sridharan, Nikhil Kesarkar, Raylene Dias

**Affiliations:** 1 King Edward Memorial Hospital Seth Gordhandas Sunderdas Medical College Department of Paediatric Anaesthesia, Mumbai, India

**Keywords:** Caudal block, child pain, hypospadias, pudendal nerve block, regional

## Abstract

**Objective:**

Postoperative pain control after hypospadias surgery can be challenging, and the effectiveness of caudal block (CB) for analgesia is limited. This study evaluated the analgesic efficacy of pudendal nerve block (PNB) using both ultrasound and a peripheral nerve stimulator (PNS), compared to a CB performed using landmark guidance, in paediatric patients undergoing hypospadias surgical repair.

**Methods:**

A total of 40 patients scheduled for hypospadias surgery were included in this prospective, randomized, double-blind controlled trial, who received either a PNB or a CB. Patients in the pudendal group received an ultrasound- and PNS-guided, PNB with a combination of bupivacaine (0.25%) at a dose of 0.5 mL kg^-1^ and clonidine at a dose of 1 µg kg^-1^, whereas those in the caudal group received a landmark-guided CB with bupivacaine (0.25%) at a dose of 1 mL kg^-1^ along with clonidine at a dose of 1 µg kg^-1^. The objective pain scale (OPS) was used to assess pain intensity in each group within 24 hours post-surgery. Perioperative hemodynamic changes and analgesic requirements were also recorded.

**Results:**

The CB provided effective analgesia, lasting an average of 6 hours. OPS scores at 6, 12, 18, and 24 hours after surgery were significantly lower in the PNB group than in the CB group. The PNB group had a significantly longer time to the need for initial analgesia, while the CB group required a significantly greater dose of paracetamol after surgery (*P* < 0.001).

**Conclusion:**

Findings from this study suggest that, at these doses, PNB is more effective than CB in providing longer-lasting pain relief, significantly lower pain scores, and a reduced need for postoperative analgesics.

**Main Points**

• Postoperative pain is a notable concern in hypospadias surgery.

• Pudendal nerve block provided more effective and longer-lasting postoperative analgesia, resulting in significantly lower pain scores compared to caudal block in the first day post-surgery.

## Introduction

Hypospadias, a prevalent congenital malformation of the penis, affects approximately 1 in 300 live infants. This condition results from developmental anomalies of the foreskin, penile urethra, and the ventral region of the penis during the embryonic stage.^1^

Early surgical intervention improves both functional and cosmetic outcomes, but it often leads to significant postoperative pain that is inadequately managed by systemic analgesics.^2^

Regional anaesthesia, particularly caudal block (CB) and penile block, plays a crucial role in pain control during paediatric genital surgeries. While CB is easy to administer and reduces the need for intraoperative analgesia, it can cause complications such as penile engorgement, impaired wound healing, and fistula formation.^3^ Similarly, penile block may result in hematoma, swelling, or inconsistent pain relief.^4^ Recently, pudendal nerve block (PNB) has been growing in popularity as a preferred alternative, providing effective intraoperative and prolonged post-surgical analgesia with reduced adverse effects.^5^^, ^^6^ Therefore, we carried out a double-blind, randomized controlled trial with a prospective study design to compare the analgesic efficacy and duration of analgesia of PNB with CB in paediatric patients undergoing hypospadias surgery. We hypothesized that PNB would offer superior outcomes compared with CB. This study primarily focused on comparing postoperative pain levels between the two groups using the objective pain scale. The secondary objectives included assessing hemodynamic changes during surgery and the analgesic requirements for both groups.

### Inclusion Criteria

Paediatric patients between 1 and 7 years old, with ASA grade 1 or 2 undergoing elective hypospadias surgical repair.

### Exclusion Criteria

Patients with a known allergy to local anaesthetics, coagulation disorders, bleeding tendencies, infections or rashes at the injection site, spinal deformities, or neurological impairments were excluded.

## Methods

This research followed a double-blind, randomized controlled trial methodology with authorization from the Institutional Ethics Committee of King Edward Memorial Hospital Seth Gordhandas Sunderdas Medical College (approval no.: EC/OA-186/2022, date: 27.02.2023). This trial was officially registered with the Clinical Trials Registry-India (CTRI/2023/06/053651). In the pre-anaesthesia assessment clinic, forty paediatric patients, ranging in age from 1 to 7 years, scheduled for elective surgery for hypospadias, were recruited a day before surgery. Written informed consent was obtained from parents for their children’s participation in the study. A comprehensive pre-anaesthetic evaluation, including a detailed medical history, physical examination, and necessary investigations like complete blood count, urine analysis, and microscopy was done. A computer-generated random number technique was used, and patients were allocated randomly to 2 groups, each consisting of 20 participants: Group CB and Group PNB. To maintain blinding, the allocation sequence was placed in sealed envelopes. The anaesthesiologist, who was not part of the study, opened the envelope and was aware of the group assignment. However, the physician collecting postoperative data and the patient were kept unaware of the group assignment, ensuring the study remained double-blinded.

After confirming adequate NPO status, all children were administered oral midazolam of 0.75 mg kg^-1^ mixed with a fruit-flavoured (watermelon) clear solution, 30 minutes before induction in the preoperative area. During this time, monitoring included vital signs, sedation level, and behavioural responses.

Patients were then transferred to the operating room, where they were connected to standard ASA monitors, such as a blood pressure cuff (non-invasive), ECG and pulse oximeter, and their baseline values were recorded. Induction was performed using a face mask with a 50:50 mixture of oxygen and nitrous oxide at 6 L min, along with incremental sevoflurane (2% to 8%), with the patient breathing spontaneously. Once a sufficient depth of anaesthesia was achieved, an intravenous (IV) line was secured, and an initial infusion of balanced electrolyte solution containing 1-2.5% dextrose was administered at a rate of 10 mL kg^-1^ h^-1^, with subsequent adjustments based on the patient’s needs. An i-gel^®^ (Intersurgical, Berkshire, UK), a second-generation supraglottic airway device, was placed following the manufacturer’s instructions. Anaesthesia  was maintained with spontaneous ventilation using a 50:50 mixture of oxygen and N₂O, with end-tidal sevoflurane regulated to 1 MAC, corrected for the patient’s age.

### PNB group

Patients were placed in the lithotomy position, followed by cleaning and draping of the surgical site. Injection sites were identified at the 3 and 9 o’clock positions, approximately two to three centimetres from the centre of the anus. A transverse ultrasound scan, using a curvilinear transducer with a frequency range of 2-5 MHz, was performed to visualize the ischium along the lateral edge of the sciatic notch. With caudal movement of the probe, the ischium aligned with the ischial spine, exposing the pudendal artery and nerve, both positioned medially to the spine. A 22-24-gauge stimuplex A nerve stimulator needle (50-100 mm; B. Braun, Melsungen, Germany) was utilized to stimulate the nerve, allowing for the observe  contractions. The needle was inserted just medial to the ischial tuberosity, at a right angle to the skin, and passed through the sacrotuberous ligament. The stimulation current was decreased to 0.5-0.6 mA by adjusting the needle position to maintain muscle contractions. After confirming no blood aspiration, 0.5 mL kg^-1^ of 0.25% bupivacaine, along with 1 µg kg^-1^ of clonidine was injected.^7^^, ^^8^

### CB group

Patients allocated to this group were positioned laterally, and with the aid of a landmark-based approach, after preparing and draping the area, a 22-gauge needle was directed into the sacral hiatus at a 45° angle. Following a negative blood aspiration, after reaching the sacral epidural space, 1 mL kg^-1^ of 0.25% bupivacaine combined with 1 µg kg^-1^ of clonidine was injected over 30-60 seconds.^9^^, ^^10^ Once the block was completed, the patients were repositioned supine, and surgery commenced 10 minutes later.

During surgery, an elevation in mean arterial pressure or heart rate exceeding 20% from the preincision baseline was considered indicative of inadequate analgesia. In such cases, an injection of fentanyl (1 to 2 µg kg^-1^) was used to provide rescue analgesia, and these patients were labelled as block failures. The surgery duration and the time needed to complete the block were recorded. Vital signs, including heart rate, SpO₂ and mean arterial pressure, were continuously monitored. All patients remained hospitalized for 24 hours after surgery to determine the block’s effectiveness.

The OPS, formulated by Broadman et al.^11^, was utilized to assess the effectiveness of pain control. This scale evaluates five factors, namely positioning, movement, crying, mean arterial blood pressure, and agitation, each rated from 0 (none) to 2 (severe), as outlined in Table 1. A scoring scale between 0 and 10 is used, with 0 to 1 indicating adequate analgesia, 2 to 3 indicating mild pain, and scores above 3 indicating severe pain. A resident in the post-anaesthesia care unit (PACU) recorded OPS scores immediately upon the patient’s arrival and at 30-minute intervals for the first 24 hours post-surgery. Those with an OPS score falling within the range of 2 to 3 received intravenous paracetamol (15 mg kg^-1^) every 6 hours, whereas those with a score of 4 or above were given intravenous fentanyl (0.5 µg kg^-1^).

The required sample size was derived with SPSS software using the appropriate formula: n = (Zα/2 + Zβ) ² × 2 × σ²/d², setting the alpha error at 5% and power at 80%. A study conducted by Naja et al.^12^ showed that 70% of children in the caudal epidural block group needed analgesics 24 hours post-surgery, while only 20% of those in the PNB group did. The calculation determined that each group required 20 patients, making a total of 40 participants.

### Statistical Analysis

The data were analysed using SPSS version 20.0, a statistical tool created by IBM. For comparing frequencies and percentages of qualitative variables, the chi-square or Mann-Whitney test was utilized, whereas the Student’s t-test was applied for quantitative variables, with a *P* value of <0.05 indicating statistical significance.

## Results

**Figure 1:**
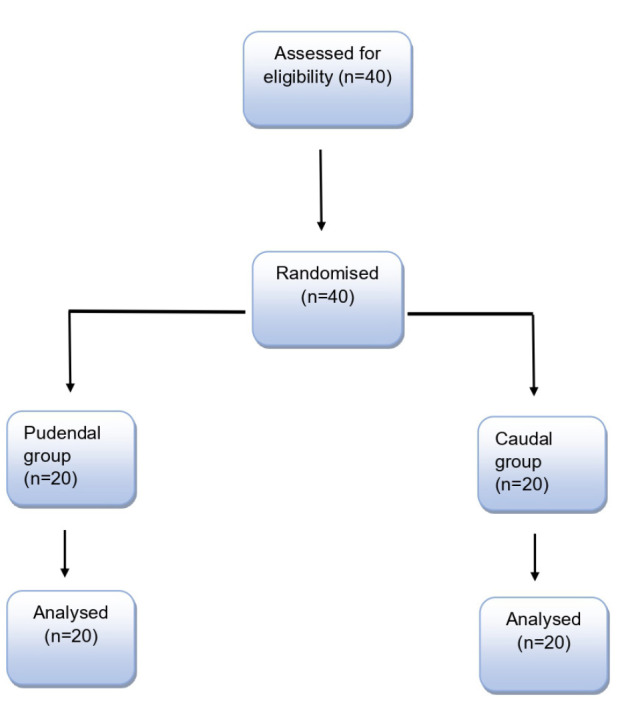
CONSORT flow diagram of the study process. After assessing eligibility, 40 patients were recruited and randomly allocated into two groups, with 20 patients assigned to the pudendal group and 20 to the caudal group, followed by subsequent monitoring and analysis.

This trial included 40 patients, who were evenly assigned into two groups of 20, with Group A receiving PNB and Group B undergoing CB (Figure 1). As shown in Table 2, two groups had similar attributes concerning age, weight, and surgical duration; however, the time taken to perform the PNB was significantly longer (*P* < 0.001). The CB was effective, with a mean time span of 6 hours. During the first 6 hours postoperatively, there was no statistically significant variation in OPS scores between the CB and PNB groups. After 6 hours, a significantly greater OPS score was observed in the CB group when compared to PNB group at 12, 18, and 24 hours after surgery (Table 3) (*P* < 0.05). No notable variations in heart rate (HR) were detected between the groups during or after surgery, and MAP remained stable throughout (Table 4). Paracetamol intake was markedly higher in the CB group, with a *P* value of < 0.001.

## Discussion

In our study, we evaluated the analgesic effectiveness of two techniques, the peripheral nerve block (PNB) and the CB, for children undergoing hypospadias surgery. CBs are the most commonly used method for this procedure. We hypothesised that the PNB would provide superior analgesic control compared with the central block. The primary objective was to evaluate postoperative pain severity in both groups using the OPS. Our secondary objectives included comparing perioperative haemodynamic changes and analgesic requirements in both groups. Our findings showed that the pudendal block provided longer-lasting  analgesia in the postoperative period than the CB.

**Figure 2:**
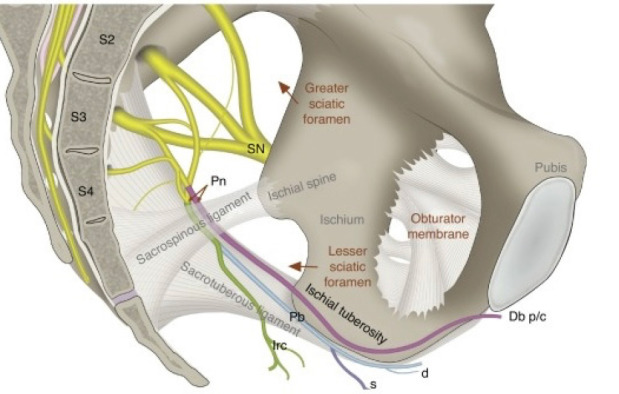
The pudendal nerve’s course in relation to the bony landmarks. Pudendal nerve (Pn), originating from the S2-S4 nerve roots, branches into the dorsal nerve of the penis/clitoris (DBp/c), the perineal nerve (Pb) with its deep (d) and superficial (s) branches, and the inferior rectal nerve (Irb). These branches are anatomically related to the sacrospinous and sacrotuberous ligaments, the greater and lesser sciatic foramina, the ischial spine, and the sciatic nerve (SN). These bony landmarks are critical for accurate localization during the block.

Arising from the second, third, and fourth ventral sacral rami, the pudendal nerve travels through the pudendal canal alongside the inferior rectal nerves before branching into the perineal nerve and the dorsal nerve of the penis.^13^^, ^^14^ Providing sensory and motor input, the pudendal nerve innervates the perineum and the penis, the primary site affected in hypospadias surgery. PNB offers comprehensive pain relief by directly targeting the pudendal nerve, along with its perineal branch, which supplies the ventral region of the penis (Figure 2). This ensures localized perineal anaesthesia and minimises systemic effects, in contrast to CB, which covers a broader area, including sacral and lumbar regions. The use of a specific method results in a reduced risk of hemodynamic instability or respiratory compromise, compared to a CB, which has a higher potential for systemic absorption, leading to a small risk of hemodynamic changes. PNB preserves motor function in the lower extremities due to targeted blockade, unlike CB, which may cause transient lower limb weakness due to involvement of sacral motor fibers. Therefore, the pudendal block is a more precise and appropriate peripheral block for targeted anaesthetic applications such as hypospadias surgery.^15^ However, there can be some sparing at the base in cases of penoscrotal hypospadias, as this area is innervated by the ilioinguinal nerve (L1 nerve root). Figure 2 illustrates the pudendal nerve course in relation to the bony landmarks. Naja et al.^12^ were the earliest to study the utilization of the pudendal block in paediatric patients undergoing surgical correction for hypospadias and circumcision. Traditionally, pudendal blocks were performed via blind injections, but techniques such as nerve stimulators, fluoroscopy, and CT scans have since been developed to guide the procedure. Our method of administering the pudendal block differed from that used in previous studies.^12^^, ^^16^ We employed a combined approach using ultrasound guidance combined with peripheral nerve stimulator (PNS), whereas earlier studies used either ultrasound or a nerve stimulator alone. The drawback of using ultrasound alone is the difficulty in clearly identifying the pudendal nerves or vessels, which complicates locating an accurate endpoint for the needle. A nerve stimulator provides a clear endpoint, such as penile bobbing or the anal wink, which improves precision. Therefore, we used a combined approach. We employed a transverse plane ultrasound to visualize the lateral aspect of the ischium within the sciatic notch, using a 2-5 MHz curvilinear transducer. Sliding the probe downward enabled clear visualization of the ischial spine, along with medial identification of the pudendal artery and nerve, ensuring accurate guidance for different age groups.^17^^, ^^18^

CB is widely regarded as a reliable and safe method for infraumbilical surgeries in children, including hypospadias surgery. However, it is associated with various adverse effects, including motor paralysis, block failure, retention of urine, and the risk of intravascular injection.^19^ Additionally, CB becomes unfeasible in cases of sacral anatomical variations or in patients with coagulopathies. More recently, several researchers have implicated CB in the development of urethral fistulas following hypospadias repair surgeries.^20, 21, 22, 23^ The proposed mechanism involves a vascular tone reduction which results in engorgement of the penile venous sinuses and excessive microvascular oozing from the surgical site after a CB. However, there is also conflicting evidence regarding this theory.^24^

In our study, baseline demographic characteristics were comparable between the groups, with no significant differences observed. We used 0.5 mL kg^-1^ of bupivacaine (0.25%) with clonidine (1 µg kg^-1^) for the pudendal block and 1 mL kg^-1^ of bupivacaine (0.25%) with clonidine (1 µg kg^-1^) for the CB.

**Figure 3:**
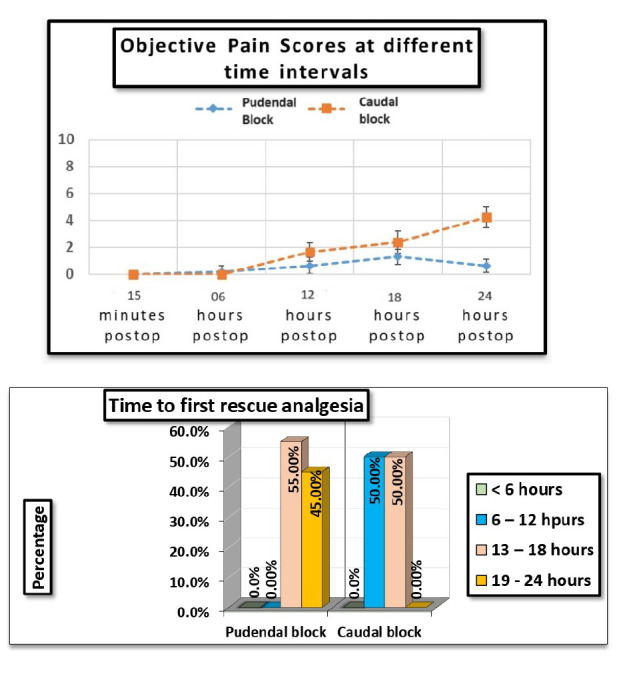
Objective pain score at different time intervals. Objective pain scores are measured at various time intervals to assess the effectiveness of interventions. X axis denotes the post operative time interval and Y axis denotes the postoperative pain severity. Objective pain score was significantly higher in caudal group than pudendal nerve block group at 6, 12, 18 and 24 hrs.

**Figure 4:**
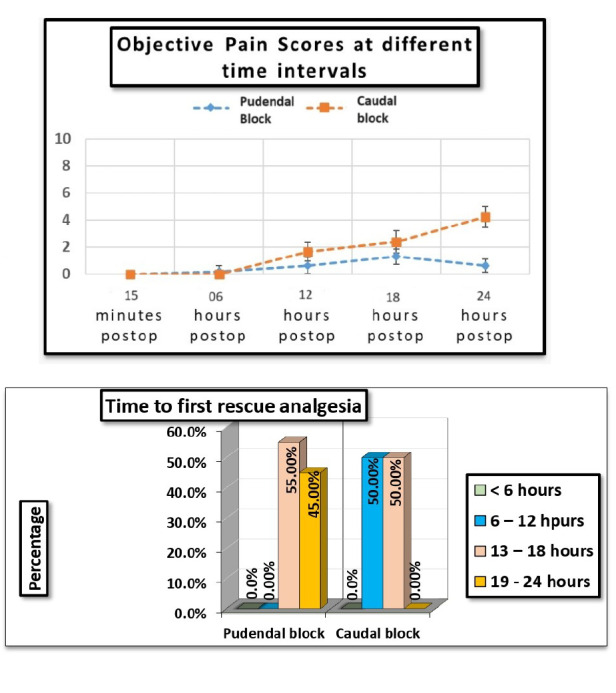
Time to administer the first rescue analgesia in 24 hours. Time to administer the first rescue analgesia within 24 hours reflects how long a patient remains pain-free after the initial block before needing supplemental pain relief. Time to first analgesia was significantly longer in pudendal nerve block group compared to caudal group.

The OPS is employed to evaluate pain and discomfort in children (8 months to 13 years of age) after procedures or surgeries by measuring behavioural changes and physiological parameters. Our investigation revealed that OPS scores were reduced in the PNB group at 12, 18, and 24 hours after surgery with *P* < 0.001 (Figure 3). The duration for the 1^st^ rescue analgesia was between 13 and 18 hours for 55% of patients in the PNB group, while the remaining 45% did not require rescue analgesia until 19-24 hours after surgery. In the CB group, Unlike the PNB group, 50% of patients required their first dose of rescue analgesia within 6-12 hours after surgery (Figure 4). Thus, PNB proved more effective in providing denser analgesia, decreasing the need for analgesic medications, and prolonging pain-free duration after surgery. The superior performance of PNB can be attributed to the administration of the anaesthetic directly at the target location, adjacent to the nerve, unlike the CB, which is a central neuraxial technique. Administering the anaesthetic near the target nerve avoids distortion of surgical planes and prevents complications such as penile engorgement or erection, which are sometimes observed after CB.

Performing an ultrasound-guided PNB with PNS requires more time than a CB due to the need for precise anatomical visualization and accurate needle placement. Performing the block in the PNB group took considerably more time, averaging 22 minutes, while the CB group achieved completion in only 6 minutes (*P* < 0.001). This prolonged duration is primarily due to the extra time required for proper positioning, particularly in older children. However, the authors believe that approximately 15 minutes of extra operating room time is justified by the superior quality and extended duration of pain relief after surgery (18-24 hours) offered by PNB, as evidenced by notable variations in OPS scores and the timing of the first rescue analgesia between the groups.

None of our patients showed signs of motor block during PACU assessment, and no technical difficulties, major complications, or neurological issues arose during the procedure. Three patients experienced transient faecal incontinence following PNB, though follow-up assessments showed no signs of anal sphincter motor dysfunction. While transient sphincter dysfunction can occur with PNB, it did not persist into the postoperative period, and no postoperative sphincter dysfunction or faecal incontinence was observed.

We successfully administered PNB in all patients, likely due to the combined ultrasound and PNS technique. Previous studies using either ultrasound or a nerve stimulator alone have reported a higher incidence of block failure.^25^^, ^^26^ Most previous studies administering PNB have focused on children ranging in age from 6 months to 2 years or from 2 to 6 years.^27^ These studies found no notable variation in the quality or duration of the analgesia between PNBs and continuous blocks in children under 2 years. However, our findings indicated that the PNB group had markedly lower OPS scores and longer-lasting analgesia than the CB group, even in children under 2 years. At 12, 18, and 24 hours after surgery, the OPS scores were significantly lower in the PNB group compared to the CB group (*P* < 0.001, subgroup analysis, Table 3).

A notable prolongation in the time to the first administration of rescue analgesia was observed in the PNB group compared to the caudal group (*P* < 0.001).

### Study Limitations

There were only a few patients in each group (only 20). Further randomized controlled studies with larger sample sizes are needed to measure pain and compare the efficacy of the two analgesic techniques. Also, we did not record surgeon satisfaction regarding variations in the surgical field, and post-operative surgical complications like fistulas. Overall, this study serves as a preliminary exploration, providing a foundation for future research with larger cohorts to validate and expand upon these findings.

## Conclusion

The results demonstrate that PNB is effective for a longer time than CB at these selected doses with significantly decreased pain scores and a reduced need for analgesics after surgery.

## Ethics

**Ethics Committee Approval:** Ethical approval was obtained from the Institutional Ethics Committee of King Edward Memorial Hospital Seth Gordhandas Sunderdas Medical College (approval no.: EC/OA-186/2022, date: 27.02.2023).

**Informed Consent:** Written informed consent was obtained from parents for their children’s participation in the study.

## Figures and Tables

**Table 1. Objective Pain and Discomfort Scale Used to Assess Pain Control (Naja et al.12, 2013):** 

**Parameter**	**Score 0**	**Score 1**	**Score 2**
1) Blood pressure	Within ±10% of preoperative value	Exceeds preoperative value by >20%	Exceeds preoperative value by >30%
2) Crying	No crying	Crying but calms with comforting	Crying and does not respond to comforting
3) Movement	No movement	Restless	Vigorous thrashing
4) Agitation	Calm or asleep	Mildly agitated	Extremely agitated
5) Posture	Normal posture	Flexing legs & thighs	Holding hands to the neck
Pain score	Interpretation		
0 to 1	Adequate analgesia		
2 to 3	Mild pain		
≥4	Moderate to severe pain		

**Table 2. Data on Patient Demographics and Clinical Details:** 

-	**Group A** **Pudendal nerve block** **(Mean ± SD)**	**Group B** **Caudal block** **(Mean ± SD)**	**Unpaired t-test value**	***P* value**
Age (in years)	4.38±1.91	3.45±1.68	1.627	0.112
Weight (kg)	14.49±3.35	12.63±2.66	1.951	0.058
Duration of surgery (minutes)	190.5±45.1	192.0±42.38	-0.108	0.915
Procedure duration to administer the block (min)	22.25±4.99	6.40±1.23	13.783	<0.001
Time elapsed until first analgesic requirement (hours)	20.30±3.51	12.15±3.00	7.897	<0.001
Subgroup analysis of children up to 2 years of age				
Time elapsed until first analgesic requirement (hours) in children aged ≤2 years	19.5±3.00	9.25±1.893	5.779	<0.001

SD, standard deviation

**Table 3. Assessment of Pain and Discomfort Progression Through Objective Scale Scores in Group A (PNB) and Group B (CB):** 

-	**Pudendal nerve block Group A** **(Mean ± SD)**	**Caudal block** **Group B** **(Mean ± SD)**	**Cohen d test value**	***P* value**
15 mins after the operation	0	0	0.0	-
6 h after the operation	0.20±0.41	0.1±0.2	0.52	0.066
12 h after the operation	0.65±0.59	1.65±0.67	1.26	<0.001
18 h after the operation	1.30±0.57	2.40±0.82	2.58	<0.001
24 h after the operation	0.65±0.49	4.25±0.79	3.26	<0.001
Subgroup analysis of children up to 2 years of age				
15 mins after the operation	0	0	0	-
6 h after the operation	0	0	0	-
12 h after the operation	0.75±0.43	2.25±0.433	3.464	<0.001
18 h after the operation	1.75±0.43	2.5±0.5	1.603	<0.001
24 h after the operation	1.25±0.43	3.25±0.433	4.618	<0.001

SD, standard deviation; PNB, pudendal nerve block; CB, caudal block

**Table 4. Assessment of Perioperative Changes in Heart Rate and Blood Pressure Conducted Between the Two Studied Groups:** 

-	**Group A** **Pudendal nerve block** **(Mean ± SD)**	**Group B** **Caudal block** **(Mean ± SD)**	**Test value**	***P* value**
1) Heart rate (beats per min) - Preoperative - Intraoperative - Postoperative	101.05±9.51 98.85±9.82 100.1±8.05	105.55±8.86 105.10±8.65 107.85±8.69	1.548 2.136 2.926	0.130 0.039 0.006
2) Mean arterial pressure (mmHg) - Preoperative - Intraoperative - Postoperative	57.5±2.52 57.4±3.53 59.15±3.58	55.85±3.13 55.5±4.32 58.13±3.97	1.834 1.523 1.667	0.074 0.136 0.104

SD, standard deviation
